# Co-Occurrence of ANCA-Associated Vasculitis and Sjögren’s Syndrome in a Patient With Acromegaly: A Case Report and Retrospective Single-Center Review of Acromegaly Patients

**DOI:** 10.3389/fimmu.2020.613130

**Published:** 2020-12-21

**Authors:** Philipp S. Fuchs, Jonas Lötscher, Caroline M. Berkemeier, Julia R. Hirsiger, Adhideb Ghosh, Quan-Zhen Li, Nikolaus Deigendesch, Emanuel Christ, Alexander A. Navarini, Mike Recher, Thomas Daikeler, Ingmar A. F. M. Heijnen, Christoph T. Berger

**Affiliations:** ^1^ Clinical Immunology, Medical Outpatient Unit, Department of Internal Medicine, University Hospital Basel, Basel, Switzerland; ^2^ Immunobiology Laboratory, Department of Biomedicine, University Hospital Basel, Basel, Switzerland; ^3^ Medical Immunology, Laboratory Medicine, University Hospital Basel, Basel, Switzerland; ^4^ Translational Immunology, Department of Biomedicine, University of Basel, Basel, Switzerland; ^5^ Faculty of Medicine, University of Zurich, Zurich, Switzerland; ^6^ Competence Center Personalized Medicine University of Zurich, Swiss Federal Institute of Technology (ETH), Zurich, Switzerland; ^7^ Department of Immunology/Internal Medicine and IIMT Microarray Core Facility, University of Texas Southwestern Medical Center, Dallas, TX, United States; ^8^ Institute of Pathology, University Hospital Basel, Basel, Switzerland; ^9^ Centre for Neuroendocrine and Endocrine Tumours, University Hospital Basel, Basel, Switzerland; ^10^ Department of Dermatology, University Hospital of Basel, Basel, Switzerland; ^11^ Immunodeficiency Clinic and Laboratory, Departments of Internal Medicine and Biomedicine, University Hospital Basel, Basel, Switzerland; ^12^ Rheumatology Clinic, University Hospital Basel, Basel, Switzerland

**Keywords:** acromegaly, Sjögren, ANCA, microscopic polyangiitis, small vessel vasculitis, whole exome sequencing, PTPN22, autoantibodies

## Abstract

**Background:**

ANCA-associated vasculitis (AAV) and Sjögren’s syndrome (SS) are uncommon autoimmune diseases. The co-occurrence in the same patient has been rarely described. Acromegaly has been associated with autoimmune thyroiditis, but the prevalence of other autoimmune disorders such as AAV and SS has not been evaluated in acromegaly.

**Methods:**

Characterization of a patient with acromegaly and two rare autoimmune diseases—SS and AAV (microscopic polyangiitis (MPA))—by autoantibody-array and whole exome sequencing (WES). Single-center retrospective review of medical records of acromegaly patients to explore the prevalence of diagnosed autoimmune diseases.

**Results:**

We report a Caucasian woman in her 50’s with a serologically (anti-SSA/Ro, anti-MPO-ANCA antibodies) and histologically confirmed diagnosis of symptomatic SS and MPA. SS with MPO-ANCA positivity preceded MPA. An exploratory autoantigen array detected a broad spectrum of autoantibodies. WES revealed heterozygous carrier status of the PTPN22 mutation R620W, which is associated with an increased risk for autoimmunity. A similar combination of positive anti-SSA/Ro autoantibodies and ANCA was only present in 5/1184 (0.42%) other patients tested for both antibodies in our clinic over six years. Amongst 85 acromegaly patients seen at our clinic in a 20-year period, 12% had a clinically relevant associated immunological disease.

**Conclusion:**

We present a rare case of SS and AAV in a patient with acromegaly and multiple autoantibody specificities. Patients with SS and ANCA should be closely monitored for the development of (subclinical) AAV. Whether acromegaly represents a risk for autoimmunity should be further investigated in prospective acromegaly cohorts.

## Introduction

Diagnosing autoimmune diseases can be challenging. The clinical presentation combined with the detection of disease-associated autoantibodies and histopathological findings of affected tissue typically allows an integrated diagnosis. Anti-neutrophil cytoplasmic antibodies (ANCA) in ANCA-associated vasculitis (AAV) and anti-nuclear antibodies (ANA) in connective tissue diseases are typical examples of disease-associated autoantibodies. However, the presence of autoantibodies is not always associated with clinical autoimmune disease (1). On the other hand, multiple autoantibody specificities may be detected in a patient with autoimmune disease, and two or more autoimmune disorders may even occur in the same patient (2). Susceptibility to autoimmune conditions has been linked to genetic variants. Examples include mutations in the protein tyrosine phosphatase, non-receptor type 22 (PTPN22) gene (3), or the NALP gene encoding for the NACHT leucine-rich-repeat protein 1 (NLRP1) (4). PTPN22 encodes for a protein tyrosine phosphatase that regulates T cell and B cell activity. NLRP1 is an intracellular sensor protein important for inflammasome formation.

Acromegaly is a rare disease with an estimated annual incidence of 2–4/1,000,000. Excessive growth hormone (GH) and insulin-like growth factor-1 (IGF-1) secretion, typically caused by a pituitary adenoma, characterize the disease. GH excess may induce coarsening of facial features, extremities enlargement, diabetes, hypertension, sleep apnea, polyarthralgia, thyroid hyperplasia with nodules, and intestinal polyps. Various immune cells, including B cells, express GH receptors (GHR) (5). The influence of GH on the immune system has however not been extensively studied. Reports on autoimmune diseases in acromegaly patients are sparse, and mostly limited to thyroid autoimmunity (6).

Here, we describe a patient with acromegaly who developed two rare autoimmune diseases accompanied by multiple autoantibody specificities. The co-occurrence of three rare diseases in the same patient prompted us to explore whether autoimmune diseases are frequent in a retrospective acromegaly cohort.

## Results

### ANCA-Associated Vasculitis, Sjögren’s Syndrome, and Multiple Autoantibodies in a Patient With Acromegaly

**Figure 1:**
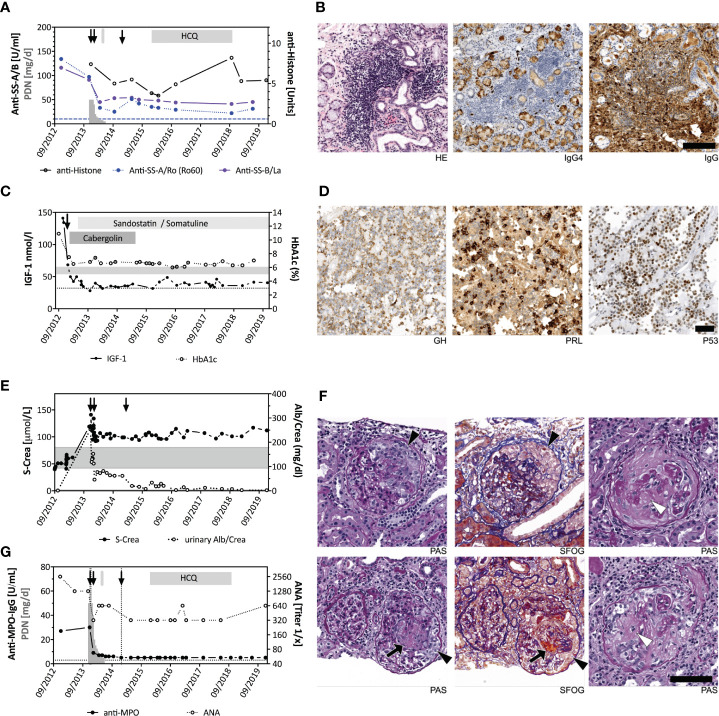
Longitudinal clinical and histopathological findings. **(A)** Time course of anti-SSA/Ro (blue), anti-SSB/La (purple), and anti-histone (black) serum levels. Timepoints of RTX treatments (arrows), prednisone (PDN) doses (gray bars indicating daily dose in mg on left y-axis), and hydroxycholoroquine (HCQ) administration is indicated. Blue dotted line represents the cut-off value of anti-SSA/B. **(B)** Labial salivary glands biopsy with hematoxyline and eosine (HE) staining reveals focal lymphocytic sialadenitis (left panel). Anti-IgG4 immunohistochemistry indicates only few IgG4 positive cells (brown) amongst the inflammatory cell infiltrate (middle panel), which was mostly IgG positive (right panel). Scale bar always indicates 100 μm. **(C)** IGF-1 (closed circles) and HbA1c (open circles) serum levels in relation to the adenoma surgery (arrow) and drug therapy of growth hormone excess (gray bars indicating duration of cabergoline and sandostatine/somatuline therapy). Dotted line = normal range of IGF-1; gray shaded area = normal range of HbA1c. **(D)** Immunohistochemistry of the pituitary adenoma shows adenoma cell positivity (brown) for GH (left), prolactin (PRL, middle) and a high expression of p53 (right). **(E)** Kidney function (serum creatinine (s-crea, closed circles) and microalbuminuria (Alb/Crea, open circles) are shown as markers of kidney disease activity. Gray shaded area = normal range for s-crea; reference for Urine-Alb/Crea is <2.6 mg/dl. Arrows indicate administration of RTX therapy. Dotted line indicates the cut-off value of MPO-ANCA. **(F)** Segmental extracapillary pauci-immune glomerulonephritis with glomerular loop necrosis (arrow), cellular crescent (arrowhead) and segmental sclerosis (open arrow head) in renal biopsy (periodic acid-Schiff stain (PAS) and SFOG staining (acid fuchsin Orange G). **(G)** MPO-ANCA (closed circles) and ANA titers (open circles) are shown.

In 2012, a woman in her 50’s with newly-diagnosed, insulin-dependent difficult-to-treat diabetes (HbA1c 14% (reference <6%)) and hypertension was referred to our clinic. She reported a two-year history of mild-to-moderate myalgia, polyarthralgia, morning stiffness (>1 hour), and oral sicca. She had observed a slowly progressive swelling of the hands and feet, and more recently headaches. At age 22, she had a partial thyroidectomy for goiter. Clinical examination showed no synovitis nor neurological deficits. Cervical and axillary lymph nodes were enlarged. CT scan revealed generalized lymphadenopathy. There was no evidence for thymoma. No pathogen could be identified as a potential cause of lymphoproliferative disease. ANA were strongly positive (titer 1:2560; reference <1:40) with anti-SSA/Ro and anti-SSB/La reactivity (**Figure 1A**; **Table S1**). Total serum IgG was elevated (20.5 g/l, reference 7.0–16.0 g/l), with increased polyclonal IgG1, IgG2, and IgG4. Schirmer’s test (without topical anesthetics) were abnormal (3 mm (left eye) and 5 mm (right eye) (reference >10 mm)). Ocular staining scores and saliva secretion quantification were not assessed. Salivary gland biopsy of the lip confirmed primary Sjögren’s syndrome (SS) (**Figure 1B**). Diagnostic resection of an axillary lymph node showed non-specific B cell proliferation (for a detailed description, see **Supplementary Material**). Anti-citrullinated protein antibodies (ACPA) and rheumatoid factor were not detectable. Radiograph of the extremities showed no erosions, but soft tissue thickening, as typically observed in acromegaly. Serum levels of IGF-1 were markedly elevated (**Figure 1C**). Brain MRI showed a pituitary tumor; transsphenoidal resection was performed. Histology revealed a growth hormone and prolactin positive pituitary adenoma (**Figure 1D**). Post-surgery, the serum levels of IGF-1 remained slightly elevated. The swelling of the extremities decreased, and arthralgia subsided. Glycemic control improved, and insulin therapy was stopped. Lymphadenopathy and sicca symptoms persisted. MRI three months post-surgery suggested the presence of remaining tumor tissue. GH suppressive treatment with cabergoline, followed by synthetic somatostatin was started. At that time, the patient was diagnosed with hyperthyroidism due to a toxic multinodular goiter. She was treated with carbimazole, and a year later with radioiodine therapy.

One year following acromegaly and SS diagnosis, an asymptomatic decline of kidney function with a glomerular filtration rate decreasing from 108 to 45 ml/min/1.7 m^2^ was observed (**Figure 1E**). The patient developed proteinuria (>3 g/l) and glomerular microhematuria (20–30 erythrocytes/high-power field, 50% glomerular). P-ANCA with anti-MPO (MPO-ANCA) reactivity was clearly positive. Complement factors C3 and C4 in serum were normal, and anti-GBM antibodies could not be detected (**Table S1**). A retrospective analysis of a stored serum sample confirmed MPO-ANCA positivity at a comparable level already at the initial diagnosis of SS one year before. Kidney biopsy revealed active pauci-immune glomerulonephritis consistent with microscopic polyangiitis (MPA) (**Figure 1F**). Lung CT scan showed no pulmonary vasculitis.

In order to explore the presence of additional autoantibodies as an indication of dysregulated immunity, an experimental autoantibody microarray with 128 different autoantigens was used (7). Besides the anti-SSA/Ro and anti-MPO antibodies which had already been identified as part of the routine clinical work-up (**Table S1**), IgG antibodies against a broad spectrum of additional antigens was found in a sample taken at the time of AAV diagnosis (**Figures S1** and **S2**). In comparison to control individuals, antibodies with the highest distinction scores included anti-ssRNA, anti-PM/Scl75, and anti-Nup-62.

We treated her AAV with glucocorticoids and rituximab (RTX; 2 x 1 g). Kidney function stabilized. Microalbuminuria rapidly improved and ultimately normalized (**Figure 1E**). Sicca symptoms and lymphadenopathy completely resolved following RTX therapy. After 12 months, RTX was repeated once to maintain remission. Hydroxychloroquine was added because of recurrent arthralgia without arthritis. During follow-up of five years since the last administration of RTX, the patient remained relapse-free. MPO-ANCA became undetectable, whereas anti-SSA/Ro antibodies persisted (**Figures 1A, G**). Schirmer’s test values during long-term follow-up revealed ameliorated lacrimal gland function (5 mm (left eye) and 11 mm (right eye) (reference >10 mm)). IGF1 serum levels remained slightly elevated despite intensified therapy with lanreotide and cabergoline. MRI seven years post-surgery showed no evidence of pituitary tumor recurrence. Whole exome sequencing of peripheral blood derived DNA identified a heterozygous carrier status of the PTPN22 mutation R620W (rs2476601), an autoimmunity risk allele (3). We confirmed the PTPN22 mutation by Sanger sequencing (**Figure S3**).

### Co-existence of Anti-SSA/Ro Antibodies and ANCA

To explore the frequency of positive ANCA and anti-SSA/Ro co-existence in patients tested at our institute, we performed a retrospective database query (for details see **Figure S4**). Amongst all available laboratory information system entries (03/2013–10/2019) we identified 1185 requests for the combination of anti-SSA/Ro and ANCA in the same serum sample. Of these, four (0.34%) -including the case presented here- had both anti-SSA/Ro antibodies and MPO-ANCA, while two (0.17%) had both anti-SSA/Ro antibodies and PR3-ANCA. None of the five cases, other than the patient presented here, had acromegaly. In addition to the presented case, two others were diagnosed with SS and suspected or confirmed AAV. The remaining three had SS or another connective tissue disease, but no AAV (**Figure S4**).

### Occurrence of Immunological Diseases in Patients With Acromegaly

Acromegaly, AAV, and SS are rare conditions with an estimated annual incidence of <1, 2.2, and 8.8 per 100’000, respectively (8, 9). Mathematically, the probability that all three diagnoses occur in the same patient is 5 out of 10^11^. We, therefore, performed an additional retrospective single-center study to explore the frequency of autoimmune disease diagnoses in acromegaly patients at our center (for the methods and the flow chart, see **Figure S5**). We identified 85 subjects (53% women, median age 57.5 years (range 22–87)) with a diagnosis of acromegaly evaluated in our hospital within 1998–2018. In 10/85 (12%) an immune system-related diagnosis (defined as ‘autoimmune disease’ or ‘lymphoproliferative disorder’) was present (**Figure S5**). The immune-related diagnoses included polyarthritis (n=3), SS (n=2, including our patient), giant cell arteritis (n=2), ankylosing spondylitis with uveitis (n=1), mucosa-associated lymphoid tissue (MALT) lymphoma (n=1), and optic nerve neuritis (n=1). In 20 of the 85 patients with acromegaly autoantibody screens had been performed. Seven of the 20 had positive autoantibody, typically an isolated ANA (titer 1:80–640; reference <1:40). The diagnoses and immunological investigations in these patients are summarized in **Table 1**.

**Table 1:** Immunological diagnoses and autoantibodies in 10/85 acromegaly patients 1998–2018.

Age	Sex	Immunological diagnoses*	ANA	SSA/Ro	ANCA	Others
51	W	AAV; SS	1:320	+	+	(+) SSB; Histone (+) (-) dsDNA; Sm
76	W	GCA	–	ND	ND	ND
74	W	GCA	ND	ND	ND	ND
43	W	HLA-B27+ AS with uveitis	–	ND	–	(-) anti-mitochondria, smooth muscle; LKM; tTg; deamid. Gliadin
35	W	MALT-lymphoma	–	–	–	(-) RF; dsDNA; Sm; anti-mitochondria; anti-smooth muscle
53	W	Optical nerve neuritis	ND	ND	ND	ND
57	W	SS; SCLE	1:640	+		(+) SSB; U1RNP; Histone (-) dsDNA; Sm; Centromer; Scl70; Jo-1
71	W	RA, Hypogammaglobulinemia	–	ND	–	ND
74	M	Seronegative polyarthritis; MGUS	–	ND	–	(-) RF; CCP
54	M	Seropositive erosive RA	1:160	–	ND	(-)dsDNA; SSB; Sm; U1RNP; Centromer; Scl70; Jo-1

AAV, ANCA associated vasculitis; SS, Sjögren’s syndrome; GCA, giant cell arteritis; HLA, human leukocyte antigen; AS, ankylosing spondylitis; MALT, mucosa associated lymphoid tissue; SCLE, subacute-cutaneous lupus; MGUS, monoclonal gammopathy of unknown significance; RA, rheumatoid arthritis; dsDNA, double-stranded DNA; LKM, liver-kidney microsomal; Sm, smith antigen; RF, rheumatoid factor; U1RNP, ribonucleoprotein U1; Scl-70, topoisomerase I; CCP, cyclic citrullinated peptide; tTg, tissue transglutaminase; deamid, deamidated; (+) indicates detected autoantibodies; (-) indicates tested, but negative autoantibodies. *Immunological diagnoses included autoimmune, antiinflammatory and lymphoproliferative diseases.

## Discussion

It is well established that patients with specific connective tissue disease, such as SS, may have multiple autoantibody specificities. B-cell hyperactivation is a central feature of SS pathophysiology that results in excessive production of immunoglobulins and autoantibodies. In addition to the anti-MPO and anti-SSA/Ro antibodies detected during diagnostic work-up of the patient, our exploratory autoantibody microarray identified reactivity against a broad spectrum of autoantigens, such as ssRNA, PM/Scl75, and Nup-62. However, in the absence of clinical evidence for diseases associated with these autoantibodies (e.g., dermatomyositis/systemic sclerosis overlap syndrome and systemic lupus erythematosus) these were regarded as clinically irrelevant. The co-occurrence of SS with other autoimmune rheumatic diseases is also a common phenomenon, albeit that AAV is rarely found in patients with SS. The French Vasculitis Consortium recently reported seven cases of AAV and SS, and summarized 15 previously published cases with clinical glomerulonephritis and positive ANCA (10). Of note, presence of acromegaly was not reported in this study. Besides, analyses of gene polymorphisms linked to autoimmunity were not reported in this case series.

Various factors may have contributed to the pathogenesis of autoimmunity in the presented case, including SS-associated polyclonal B cell activation and the PTPN22 risk allele, but also the use of carbimazole. Non-overlapping genetic susceptibility loci have been described for pituitary adenoma (11), SS (12) and AAV (13). We have explored the protein-encoding genes in our patient by performing whole-exome sequencing. We found no previously described rare variants predicted to be damaging in pathways relevant to B cell function or innate sensors (**Table S2**). The patient was a heterozygous carrier of the PTPN22 mutation R620W (rs2476601) which is a known risk allele for various rheumatic diseases (3). A noteworthy genetic association of the PTPN22 R620W polymorphism with AAV has been reported in a meta-analysis (14). However, no consistent association could be established for SS (14), although geographical differences may exist (15–17). Whether PTPN22 R620W is associated with acromegaly has, to the best of our knowledge, not been evaluated yet.

The PTPN22 gene encodes a tyrosine phosphatase that limits T cell receptor (TCR) down-stream signaling by dephosphorylating kinases such as Lck or ZAP-70 that are critical for TCR-mediated activation. Although the single nucleotide polymorphism resulting in the R620W variant impacts this interaction, the precise mechanisms of action of PTPN22 R620W in T cells and the development of autoimmunity are complex and not completely understood (18, 19).

Our patient received the antithyroid drug carbimazole after the SS diagnosis, but before overt AAV. Of note, overt AAV is a rare adverse effect of anti-thyroid drug therapy. Our patient was diagnosed with AAV approximately 12 months after treatment with carbimazole was started, but the retrospectively determined ANCA were already detectable before carbimazole was started, indicating a drug-independent ANCA development.

Acromegaly has been associated with thyroid autoimmune disorders. Cases of autoimmune diseases in acromegaly patients have been reported (20), although a recent study on a small sample of acromegaly patients could not find differences in autoantibody prevalence between acromegaly and non-acromegaly control subjects (21). In our single-center cohort, one in eight acromegaly patients had a co-existing ‘immune system-related disease’. A mouse study recently provided evidence for IGF-1-mediated Th17/Treg dysbalance, favoring development of autoimmunity. Mice without the IGF-1 receptor on T cells showed a substantially reduced disease severity in the experimental autoimmune encephalomyelitis (EAE) model of multiple sclerosis (22, 23). Interestingly, high IGF-1 was demonstrated in labial glands of pSS patients (24). Whether IGF-1 may play a role in autoimmunity or B cell proliferation in humans remains, however, speculative. Notably, our patient developed symptoms of Sjögren’s syndrome (sicca, arthralgia) in parallel to a slowly progressive enlargement of the extremities, but presumably before diabetes became apparent.

We suggest that patients with SS and positivity for MPO- or PR3-ANCA should be closely monitored for the co-occurrence or development of (subclinical) AAV. Clinicians should be attentive if an acromegaly patient presents with symptoms potentially related to connective-tissue disease or systemic autoimmunity. The observed presence of autoimmune diagnoses in acromegaly patients should, however, be further explored in larger, prospective acromegaly cohorts.

## Data Availability Statement

The raw data supporting the conclusions of this article will be made available by the authors, without undue reservation.

## Ethics Statement

The studies involving human participants were reviewed and approved by Ethics Committee of Northwestern and Central Switzerland. Written informed consent for participation was not required for this study in accordance with the national legislation and the institutional requirements. Written informed consent was obtained from the individual(s) for the publication of any potentially identifiable images or data included in this article.

## Author Contributions

PF: case report data collection and analysis, writing of the original draft. JL: retrospective search for acromegaly patients and data analysis, manuscript review/editing. CMB: methodology for the database search, data collection, manuscript review/editing. JH: data collection, manuscript review/editing. AG: whole exon sequencing analysis, manuscript review/editing. Q-ZL: protein array analysis. ND: pathology imaging and analysis, manuscript review/editing. AN: whole exon sequencing and analysis, manuscript review/editing. MR: conceptualization, whole exon analysis, manuscript review/editing. TD: data interpretation, patient care, and manuscript review/editing. IH: database search, conceptualization, methodology, writing. CTB: conceptualization, methodology, data visualization, writing of the original draft, supervision. All authors contributed to the article and approved the submitted version.

## Funding

CTB is supported by the Margot und Erich Goldschmidt & Peter René Jacobson-Stiftung.

## Conflict of Interest

The authors declare that the research was conducted in the absence of any commercial or financial relationships that could be construed as a potential conflict of interest.
