# Supplementary material for: Co-Occurrence of ANCA-Associated Vasculitis and Sjögren’s Syndrome in a Patient With Acromegaly: A Case Report and Retrospective Single-Center Review of Acromegaly Patients
Source: Front Immunol. 2020 Dec 21;11:613130. doi: 10.3389/fimmu.2020.613130 (PMC7779546; doi:10.3389/fimmu.2020.613130)
Supplement: Supplementary file 1 [file DataSheet_1.docx]

**Co-occurrence of ANCA-associated vasculitis and Sjogren’s syndrome in a patient with acromegaly: A case report and retrospective single-center review of acromegaly patients**

Philipp S. Fuchs et al.

***Supplementary Material***

1. ***Supplementary Figures***

*Figure S1: Heatmap of the Autoantibody-Array*

*Figure S2: Principal component analysis and examples of autoantibody-array results*

*Figure S3: Sanger Sequencing of the PTPN22 R620W SNP*

*Figure S4: Flow chart of the immunological lab screen*

*Figure S5: Flow chart of the retrospective acromegaly cohort.*

1. ***Supplementary Tables***

*Table S1: Overview of longitudinal autoantibodies, immunoglobulin levels and lymphocyte subset phenotyping results*

*Table S2: Rare variants in genes related to primary immunodeficiency*

1. ***Supplementary Data***

***Supplementary Figures***

******

***Figure S1: Heatmap of the Autoantibody-Array.*** We used a multiplexed microarray with 128 autoantigens to measure autoantibody profiles ^1 2^. The antibody score was calculated by log2-transformed, background-adjusted signal intensity times signal-to-noise ratio (SNR). Columns show autoantibody scores in the patient (purple; three independent timepoints: before, one-year after and 5 years after rituximab therapy for AAV) compared to fifteen healthy controls (green) are shown. Every row represents one autoantigen. Red=increased and blue= reduced compared to row average.

**
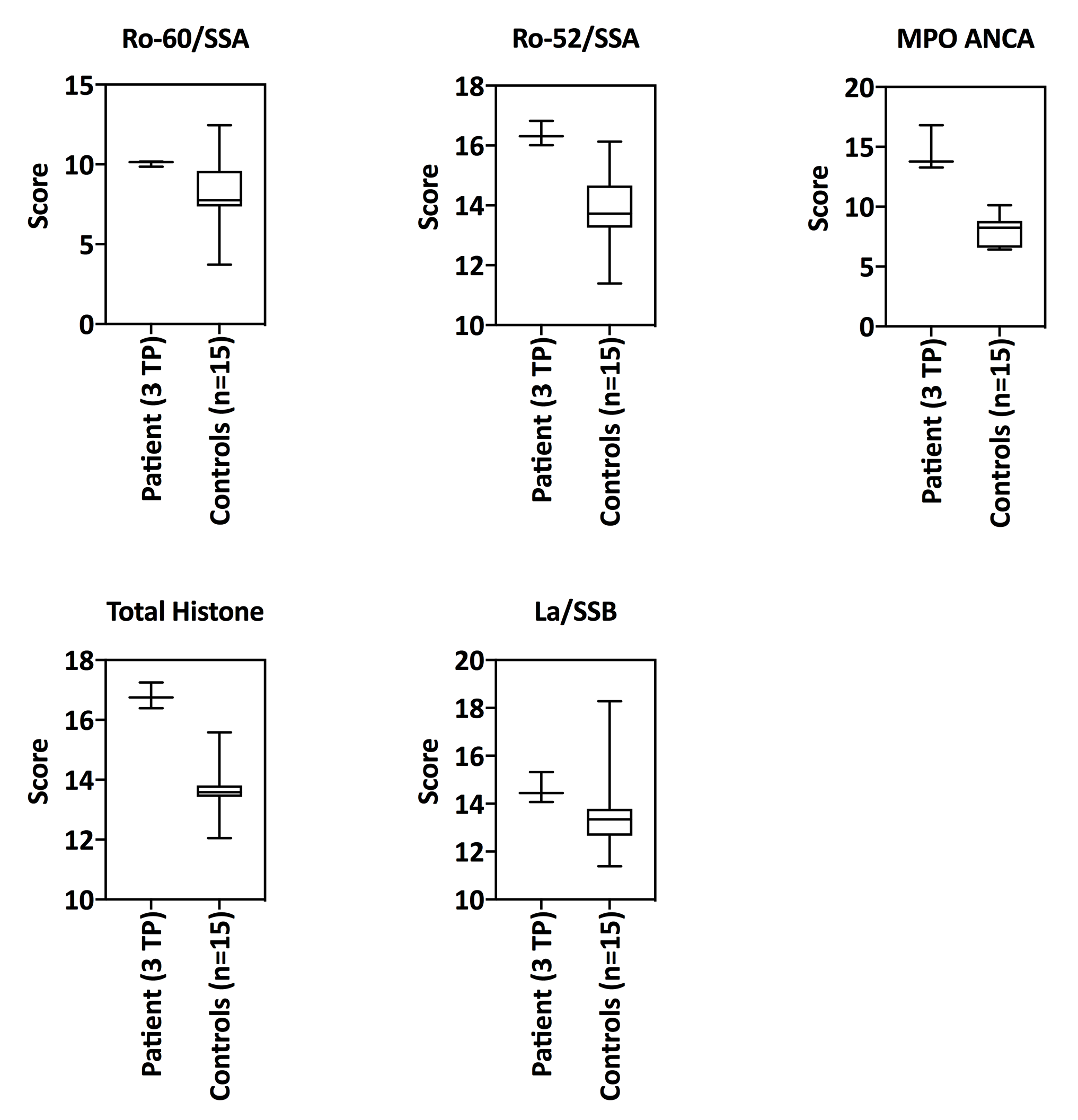
**

***Figure S2: Principal component analysis and examples of autoantibody-array results.*** Complementary analyses to Figure S1: (A) Principal component analysis (PCA) of the autoantibody scores indicates that the autoantibody profile from the three timepoints in the patient (purple; i.e. at AAV diagnosis, (07/2013), and approximately one (05/2014) and five years later (09/2018) are clearly distinguished from the profile in the 15 healthy controls (green). PCA was calculated with ClustVis. Same data as in Figure S1. (B) Box plots show the absolute autoantibody score in the patient and controls for autoantibodies that were positive in the clinical routine diagnostics. Box indicates 25^th^-75^th^ percent and line indicates the median.

***Figure S3: Sanger Sequencing of the PTPN22 R620W SNP.*** Sanger sequencing was performed to confirm the heterozygeous R620W carrier status (indicated by arrow).

***
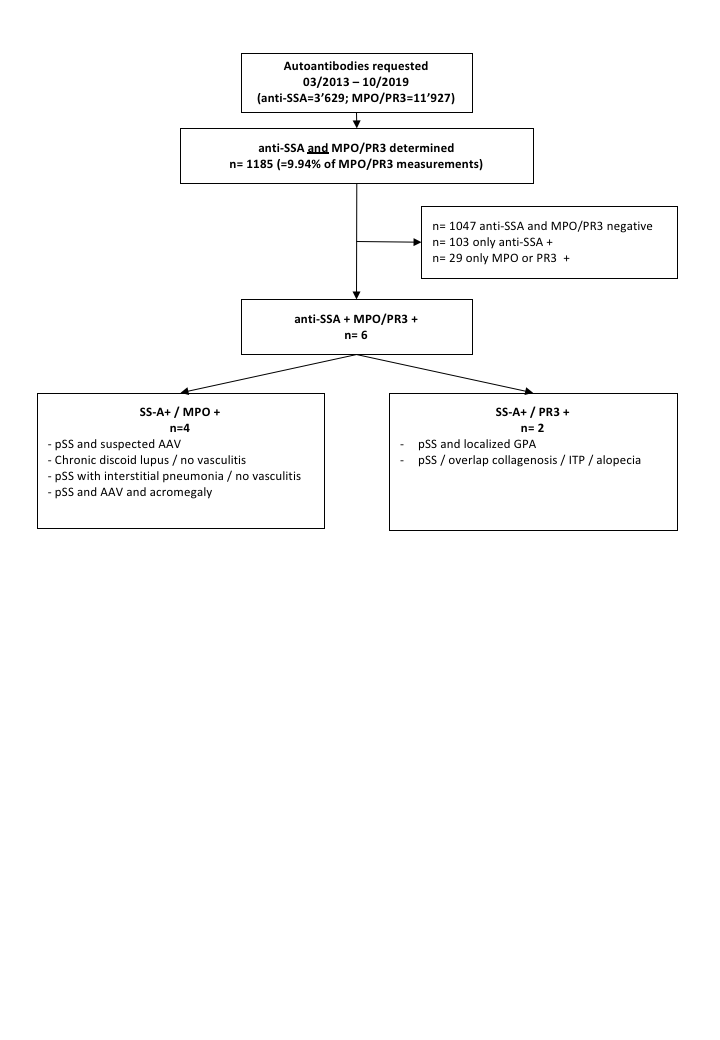
Figure S4: Flow chart of the immunological lab screen.*** The local ethics board (ID#2018-00596) approved the study. The clinical laboratory information system (LIS) was queried for autoantibody measurements in these patients. Separately, all available LIS entries (starting 03/2013) with a request for both ANCA and anti-SSA/Ro antibodies were retrieved. ANCA detection included both indirect immunofluorescence tests on ethanol-fixed neutrophils and immunoassays against MPO and PR3. Anti-SSA/Ro IgG were detected by immunoassay and – in case of positivity – subsequently tested for anti-Ro52 and anti-SSA/Ro60 positivity. In our hospital 3629 Anti-SSA and 11927 MPO-/PR3-ANCA tests were requested 03/2013-10/2019. Of those, 1185 included concomitant analysis of anti-SSA and MPO-/PR3-ANCA. Six were positive for both. All but one had a diagnosis of SS. Two had an AAV and one a suspected AAV.

***
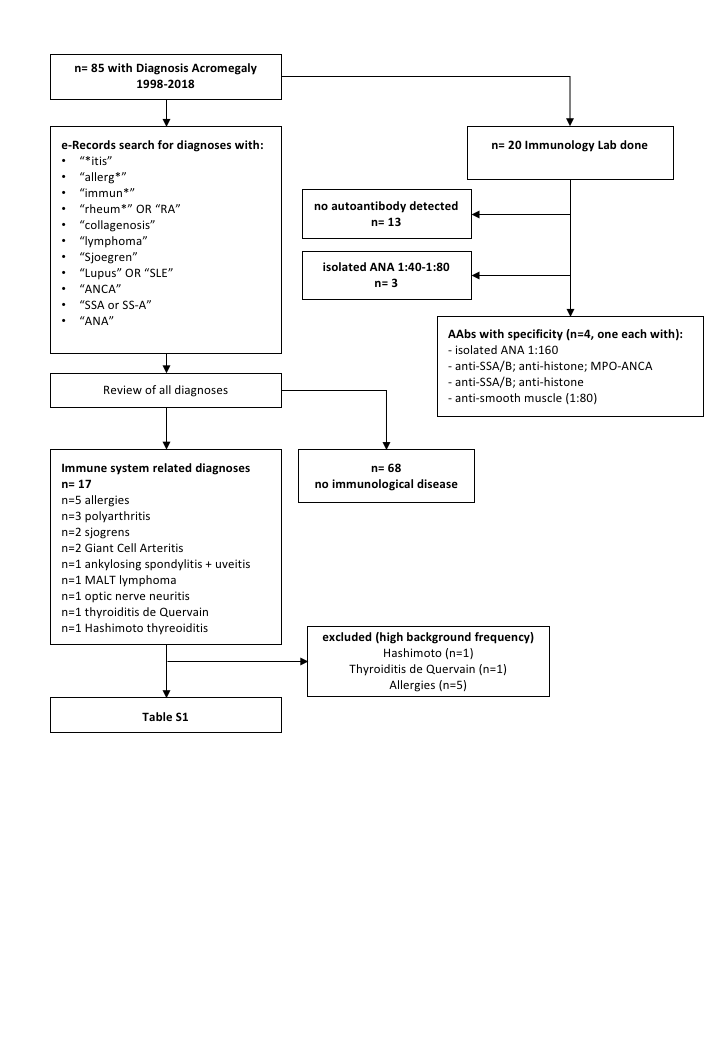
Figure S5: Flow chart of the retrospective acromegaly cohort.*** Diagnoses in the electronic charts of patients of the University Hospital Basel from 1998-2018 were screened for ‘acromegaly’. Within the acromegaly patients we identified those with autoimmune diagnoses. Relevant immunological diagnoses (excluding n=5 ‘allergies’, n=1 ‘thyroiditis deQuervain’, and n=1 ‘Hashimoto’) in acromegaly patients are summarized in ***Table S1***. Notably, the immunological diagnoses were not necessarily diagnosed during active acromegaly, but were noted during the observation period (1998-2018)

***Supplementary Tables***

***Table S1: Overview of longitudinal autoantibodies, immunoglobulin levels and lymphocyte subset phenotyping results***

******

Color code: green normal, yellow to red pathological. Pre-surgery refers to a sample previous to pituitary adenoma surgery. RTX= rituximab; mts=months; y = years

***Table S2: Rare variants in genes related to primary immunodeficiency***

| **Chr** | **Pos** | **ID** | **Ref** | **Alt** | **Gene** | **Consequence** | **Type** | **MAF gnomAD** | **PolyPhen** | **CADD Phred** |
| --- | --- | --- | --- | --- | --- | --- | --- | --- | --- | --- |
| 4 | 126370186 | rs138655269 | A | T | FAT4 | Non Synonymous | Missense | 0.002 | probably damaging (0.98) | 25.3 |
| 5 | 94863731 | n/a | G | A | TTC37 | Non Synonymous | Missense | 0 | benign (0) | 16.4 |
| 7 | 117188840 | rs377319489 | AGTT | A | CFTR | Inframe | Inframe Deletion | 0 | NA | 19.96 |

*Chr.= chromosome; pos= position; ID= SNP ID; Ref= reference nucleotide at the position; Alt= alternative nucleotide at the position; Type= type of nucleotide change; Polyphen= predicted impact on protein; CADD= Combined Annotation Dependent Depletion; score to define deleteriousness of a nucleotide change on the protein*

***3) Supplementary Data***

***Histopathology report summaries***

***Kidney biopsy*** exhibited recurrent focal and segmental extracapillary pauci-immune glomerulonephritis with fresh glomerular loop necrosis in 1/30 glomeruli, cellular crescents in 4/30 glomeruli, segmental sclerosis and fibrous crescents in 8/30 glomeruli, and slightly increased number of completely sclerotic glomeruli (4/30). Moderate focal interstitial fibrosis with tubular atrophy (40-50%). Using immunofluorescence on cryosections only small granular mesangial, partly peripheral deposits of C5-9 within glomeruli. IgA, IgG, IgM, kappa and lambda light chains, C3 and fibrinogen negative.

***Small salivary gland*** biopsy from the lower lip showed mild fibrosis and > 1 lymphocytic focus/4 mm^2^, corresponding to Chisholm and Mason grade IV. Plasma cells in the salivary gland were predominantly IgA positive, less IgG and sparse IgM. Low IgG4:IgG ratio with only few IgG-positive plasma cells.

***Lymph node biopsy*** (not shown) was stained for CD3, CD5, CD20, CD10, BCL6 and BCL2 as well as CD21 and CD23 and showed a disturbed architecture with increased B-lymphocytes, partly prominent mantle zones, widening of marginal zone and partly regressively altered secondary follicles. No presence of aberrant immunophenotype of the lymphocytes or other evidence for a neoplastic process. Ki-67-proliferation index partially increased, but within normal range. Cyclin D1 negative. Anti-CD38 staining shows a sinusoidal plasmacytosis. Plasma cells show a ratio of IgG:IgM:IgA of about 10:1:1 of heavy chain expression. Number of IgG4-positive plasma cells increased and occasionally forming small groups. Overall ratio of IgG:IgG4 about 5:1, and less than fifty IgG4-positive plasma cells per HPF detectable.

***References***

1. Li QZ, Xie C, Wu T, et al. Identification of autoantibody clusters that best predict lupus disease activity using glomerular proteome arrays. *J Clin Invest* 2005;115(12):3428-39. doi: 10.1172/JCI23587 [published Online First: 2005/12/03]

2. Perez-Diez A, Wong CS, Liu X, et al. Prevalence and pathogenicity of autoantibodies in patients with idiopathic CD4 lymphopenia. *J Clin Invest* 2020 doi: 10.1172/JCI136254 [published Online First: 2020/07/08]
